# Health, Well-Being and Energy Poverty in Europe: A Comparative Study of 32 European Countries

**DOI:** 10.3390/ijerph14060584

**Published:** 2017-05-31

**Authors:** Harriet Thomson, Carolyn Snell, Stefan Bouzarovski

**Affiliations:** 1 School of Environment, Education and Development, University of Manchester, Manchester M13 9PL, UK; stefan.bouzarovski@manchester.ac.uk; 2 Department of Social Policy and Social Work, University of York, York YO10 5DD, UK; carolyn.snell@york.ac.uk

**Keywords:** energy poverty, fuel poverty, relative deprivation, health inequalities, well-being

## Abstract

Despite growing pan-European interest in and awareness of the wide-ranging health and well-being impacts of energy poverty—which is characterised by an inability to secure adequate levels of energy services in the home—the knowledge base is largely British-centric and dominated by single-country studies. In response, this paper investigates the relationship between energy poverty, health and well-being across 32 European countries, using 2012 data from the European Quality of Life Survey. We find an uneven concentration of energy poverty, poor health, and poor well-being across Europe, with Eastern and Central Europe worst affected. At the intersection of energy poverty and health, there is a higher incidence of poor health (both physical and mental) amongst the energy poor populations of most countries, compared to non-energy poor households. Interestingly, we find the largest disparities in health and well-being levels between energy poor and non-energy poor households occur within relatively equal societies, such as Sweden and Slovenia. As well as the unique challenges brought about by rapidly changing energy landscapes in these countries, we also suggest the relative deprivation theory and processes of social comparison hold some value in explaining these findings.

## 1. Introduction

Energy poverty is typically attributed to an interaction between low incomes, energy inefficiency and energy prices [1], and may be compounded by other factors such as tenure type, age, and health status [2]. In addition to rising energy costs, the economic downturn, and cuts to public sector have all been linked—at least in passing—to an increase in energy poverty [3,4]. At the heart of most discussions of energy poverty lies concern about the affordability of energy [1,5,6,7]. This focus has been shaped by the highly influential work of Brenda Boardman [8] that conceptualised energy poverty as ‘the inability to afford adequate warmth because of the inefficiency of the home’ (which built on previous affordability research conducted by researchers such as Lewis [9]), and has subsequently been developed by academics such as John Hills [5] in the 2010s [6]. Whilst research conducted in the UK has typically focused on warmth there is also a growing literature base that, amongst other things, indicates the importance of access to energy for cooling purposes in countries with warmer climates (see for example [10,11]).

At a policy level energy poverty has been measured on the basis of the affordability of energy in the UK, and over the last decade the approach has gained resonance with other European countries, with France and Ireland adopting an affordability measure, and Slovakia and Austria being in the process of developing one. It should be noted that within some contexts, most notably in the UK, energy poverty has been recognised via the term fuel poverty, and the two terms are often used interchangeably, even within official EU policy documents [12].

Alongside a substantial body of literature that has considered the definition and measurement of energy poverty at the national [13,14,15,16] and pan-European scale [17,18,19,20], an equally large evidence base has been developed since the 1980s around the consequences of energy poverty on human health [21,22]. Knowledge has developed around the relationship between conditions typically associated with energy poverty, such as indoor air temperatures, poor housing conditions, and health. The compounding impact of other socio-economic and demographic factors, and the impact of policy responses have also been widely researched [23,24,25,26,27,28,29,30].

However, there remain substantial gaps in knowledge about the impacts of energy poverty at the European level (discussed further below). This paper aims to add to the highly limited knowledge base that currently exists, with the intention of opening new lines of discussion in what has become a widely discussed policy topic at the EU-scale, especially given on-going reviews of relevant energy legislation [12]. Indeed, the Republic of Ireland recently announced funding of €10 million for the Warmth and Wellbeing scheme, that provides free energy efficiency upgrades for households who are classified as energy poor and contain young children with chronic respiratory conditions [31]. This paper also aims to make a contribution to the growing literature on the outcomes to exposure to poor quality housing within specific populations worldwide [32,33].

It should be noted at this juncture that this paper focuses on the relationship between access to affordable warmth and health. Whilst the authors both acknowledge and recognize that the term energy poverty can be used to encompass broader issues such as access to cooling facilities [11], the limited data that exist do not allow a broader focus such as this to be taken. 

## 2. Background

The Marmot Review into the Health Impacts of Cold Homes and Fuel Poverty [27] found a strong relationship between cold temperatures and cardiovascular and respiratory diseases, in addition to links between cold housing and minor illnesses such as colds and flu. As summarised by the UK’s fuel poverty strategy in 2001: ‘*The likelihood of ill health is increased by cold homes, with illnesses such as influenza, heart disease, and strokes all exacerbated by the cold. Cold homes can also promote the growth of fungi and numbers of house dust mites. The latter have been linked to conditions such as asthma. Ill health can lead to...certain types of illness, such as respiratory disease*’ [34]. A relationship between poor mental health and experiencing energy poverty is also evident in the literature [27]. The evidence base typically focuses on anxiety, stress and depression associated with living in poor housing conditions, balancing bills, heating needs and debt [4,26,33,35,36]. Gilbertson et al. also find that cold, draught, and condensation are associated with anxiety [37], as is the cost of energy (a point also supported by Kaye et al. [3]). Early research by the World Health Organization (WHO) [38] identified both young and old age, and the presence of an existing condition or disability as factors that increase vulnerability to the effects of low temperatures. Since then substantial research has been conducted about the health impacts on these groups, and additional risk factors have also been considered (The National Institute for Health and Care Excellence for example includes pregnant women, people with mental health conditions, and those on a low income [39]). There is also a burgeoning evidence base that connects income inequality and health, which suggests a correlation between the two [40,41]. An extensive review of the literature in 2011 identified a social gradient in health, with every step ‘up the socio-economic ladder’ leading to an increase in health [40].

As outlined above, the literature suggests that low temperatures and poor housing conditions are associated with heightened health risks for those with chronic and severe illnesses, including cardiac conditions and respiratory diseases [27,42], and that health outcomes may be substantially worse for these groups [26,27,35,42,43,44,45,46,47]. One of the explanatory factors is that sedentary or ill people are less able to generate their own heat [27,43,48]. For example Parkinson’s disease restricts physical activity, which in turn slows body heat generation and conservation [27]. Whilst WHO research of the 1980s was limited in terms of considering specific conditions and air temperatures, more is now known about this. For example Osman et al. [49], Howden-Chapman [47], and Ormandy and Ezratty [50] suggest that those with chronic obstructive pulmonary disease (COPD) require a heating regime of 21 degrees centigrade [50] and where this is not met there are severe health implications, both in the short and long term. In addition to those with underlying health conditions, older people are regarded as being more vulnerable to the effects of low indoor temperatures and poor housing conditions. This relationship exists for a number of reasons: the higher likelihood of older people having underlying health conditions, less subcutaneous fat, and being in the home for longer periods (and thus needing more heat for longer) [27]. There is also evidence that ‘thermal discomfort’ can exacerbate existing conditions such as arthritis and rheumatism [26,27] and a relationship has been found between energy inefficient housing and increased rates of winter respiratory disease among older people [51]. 

There is also a significant body of literature that addresses excess winter mortality (EWM). English data from 2012–2013 indicates that only 24 per cent of cold-related deaths occurred amongst those aged under 75 [39]. Once again, a relationship between chronic and severe illnesses and EWM is suggested throughout the literature [52,53,54,55]. The UK’s Department of Health [53] highlights that those with chronic and severe illnesses such as heart conditions, respiratory insufficiency, asthma and COPD, and ‘co morbidities’ are vulnerable to EWM. While EWM figures have been consistently high in the UK and Ireland since the 1980s, countries with colder climates such as Finland, Norway and Poland have substantially lower rates [56]. Counterintuitively, the only countries with higher EWM rates are located in Southern Europe: Malta, Portugal, Cyprus and Spain. In the case of the UK and Ireland, poor energy efficiency and energy costs are given as the main driver [27], whereas in Southern Europe there are other factors, such as building fabric and lifestyle adjustments [56]. 

However, despite the growth in interest in energy poverty and its wide-ranging health and well-being impacts, the European literature base tends to be both British-centric—as a consequence of its long history of academic scholarship, practice-based responses and policy frameworks to address the issue—and populated with single-country studies. Beyond analyses of EWM rates across Europe [56,57,58], virtually no comparative research has been conducted to understand the relationship between energy poverty, health and well-being in Europe. To date the only study of this nature was a report by WHO on the environmental burden of disease associated with poor housing [59], and an older examination of the health inequalities associated with energy poverty in the EU14 Member States [60]. 

In response to these significant research gaps, this paper examines the relationship between energy poverty and: (1) general health; and (2) mental well-being, across 32 countries in Europe using 2012 micro data from the European Quality of Life Survey. However, it should be emphasised that this is an initial exploratory study, and the research is both shaped and limited by the prevailing data infrastructure. The subsequent sections of this paper describe both the research methodology and its limitations, before presenting the core results from secondary data analysis, and ending with a consideration of the findings in a European context.

## 3. Materials and Methods

### 3.1. Data

Cross-sectional data from the 2012 European Quality of Life Survey (EQLS) dataset [61] is used, covering 41,560 individuals (aged 18 years or over) from 32 European countries that are currently Member States of the European Union (EU), or are official candidates for EU membership. The EQLS is a specialist repeated cross-sectional survey that focuses on living conditions, attitudes, health and well-being throughout Europe, and has nationally representative samples of between 1000 and 3000 respondents from each country [62,63]. 

### 3.2. Variables

#### 3.2.1. Predictor Variable

As discussed in the introduction, the dominant method of defining energy poverty amongst EU countries is based around the affordability of energy. At a national policy level energy poverty measures are typically based on the relationship between household expenditure on energy, income, and in some instances heating requirements. Such policy measures of affordability are used in Ireland, the UK, France, and more recently Slovakia and Austria. One of the prevailing indictors used to capture self-reported affordability and to measure energy poverty asks whether a household can afford to keep their home adequately warm. Within the EQLS this indicator is worded as follows:

“*There are some things that many people cannot afford, even if they would like them. For each of the following things on this card, can I just check whether your household can afford it if you want it? Keeping the home adequately warm*”.

[63]

Subjective measures such as this have been criticized for their lack of consistency across respondents. For example, Hills suggests that a complex mix of cultural, generational and demographic factors influence participants’ responses [5]. There are also risks associated with potential errors of exclusion and biases [64]. Despite these criticisms this indicator (along with other subjective measures) has been widely used in comparative analyses of energy poverty across Europe [17,18,20,60,65], as well as in the analysis of energy poverty and poor health in France [21]. The popularity of this subjective variable can be attributed to both epistemological and pragmatic considerations. For researchers such as Fahmy et al. [2] subjective measures provide a more nuanced perspective of energy poverty, capturing lived experience and need in a manner that cannot be captured by objective measures [2,6]. Advocates of subjective measures also argue that they are better able to capture the wider social exclusion and material deprivation elements associated with energy poverty than formulaic expenditure-based measures [60]. Furthermore, subjective measures have also been used as proxies of energy poverty in the absence of valid and reliable expenditure data, for example, when making comparisons across jurisdictions that do not have comparable expenditure data [7].

It should also be noted that this indicator does not reflect energy demands such as cooling—an issue more common in Southern Europe [10]. As such, the indicator of energy poverty used is relatively narrow in scope. This, along with the other limitations described above, is typically associated with conducting secondary data analysis (see for example [66]). Despite these limitations, this indicator currently represents one of the few available methods of quantifying energy poverty related issues across Europe, and is the measure used within this paper (from here on the term ‘energy poverty’ relates to this measure).

#### 3.2.2. Health and Well-Being Outcomes

To assess poor health, the self-reported health status (SRH) variable available in the EQLS is used. SRH is a well-established indicator that has been used extensively in public health and epidemiological research [67,68], with respondents asked to rate their general health on a 5-point scale: “In general, would you say your health is…Very Good/Good/Fair/Bad/Very Bad” [63], where ‘very good’ is assigned a score of 1 and ‘very bad’ is scored 5. Whilst this variable is self-reported, and thus risks the possibility of recall and response biases, existing evidence suggests that SRH is a highly reliable and valid measure of health status [69,70,71], which has been found to be predictive of future health outcomes [72,73]. The SRH variable was dichotomised to create an indicator of poor health, with 1 representing bad or very bad health, and 0 representing fair, good, and very good health, in line with previous categorisations of self-assessed poor health [60,63,74].

The EQLS uses the 5-item World Health Organization Well-being Index (WHO-5) to measure subjective emotional well-being. The WHO-5 index is one of the most widely used questionnaires for assessing subjective psychological well-being and has been translated into over 30 languages [75]. The WHO-5 index is comprised of the following statements:

I have felt cheerful and in good spirits;

I have felt calm and relaxed;

I have felt active and vigorous;

I woke up feeling fresh and rested;

My daily life has been filled with things that interest me.

Each component is rated on a 6-point Likert scale, ranging from ‘All of the time’, which is allocated a score of 5, through to ‘At no time’, which is assigned a score of 0. The raw scores, which range from 0 to 25, are multiplied by 4 to give a maximum value of 100 [75]. Higher scores indicate better well-being [67,76]. As evidenced by the statements above, the WHO-5 index focuses on mental well-being rather than symptoms [77]. Despite this focus, several studies have found that the WHO-5 index offers a good screening test for depression when assessed against clinical interviews [76,77,78]. From this index, two dichotomised variables were created, firstly a flag variable for identifying respondents with an index score ≤ 50, which suggests poor emotional well-being, although not necessarily depression, and secondly for identifying respondents with a score of ≤28, which is commonly used as an indicator of likely depression [75,78].

The overall weighted prevalence rates for all of the measures operationalized in this research can be found in Table 1 below. Initial observations from this table suggest that the incidence of energy poverty, poor health and well-being are distributed unevenly across Europe. In all cases, a higher proportion of respondents report poor well-being than they do bad or very bad health. 

#### 3.2.3. Socio-Demographic Adjustment Variables

A range of socio-demographic variables were used to control for factors which may confound or mediate the relationship between health and well-being status and energy poverty, namely age of respondent at last birthday (18–24; 25–34; 35–49; 50–64; 65+ years), gender (male; female), highest educational attainment (primary or less; secondary; tertiary), and income quartiles.

### 3.3. Statistical Analysis

Mann–Whitney U tests were conducted to explore the differences in general health and WHO-5 well-being scores between groups in energy poverty and those that are not. Next the prevalence rates and rate differences were calculated for poor health and well-being among the energy poor group and overall survey populations. Logistic regression was then used to compare the same relationships adjusting for potential socio-demographic confounders. The results are presented as odds ratios with 95% confidence intervals (CI) for poor health, and poor well-being, among individuals living in energy poverty. A weighting variable (w4) was applied in all analyses to ensure that the results are representative for the population of individual countries [63]. 

## 4. Results

### 4.1. Poor SRH Status and Energy Poverty

To understand the underlying trends in poor health status, the differences in mean SRH scores between groups in energy poverty and those that are not were examined first. In all instances, the mean health score was higher for individuals in energy poverty than those who were not, indicating the former has poorer SRH health. Based on Mann–Whitney U tests, statistically significant differences in scores were found in 30 of the 32 study countries, the exceptions being Austria and Finland. 

**Figure 1:**
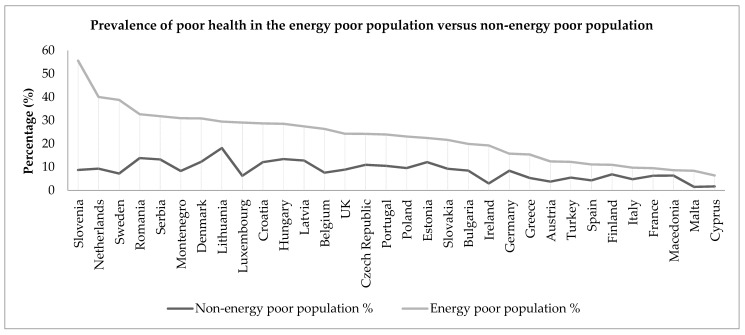
Line graph showing the prevalence of poor health among the energy poor and non-energy poor populations across 32 European countries.

In the next stage, the prevalence rates for poor health among the energy poor population and non-energy poor population were calculated, as shown below in Figure 1. From this figure, a number of observations can be made. Firstly, it is evident that poor SRH is unequally distributed across the 32 countries, with the highest overall rates of poor SRH found within Central and Eastern European countries, such as Lithuania, Romania, and Serbia. Secondly, the population of energy poor individuals living across Europe has a higher incidence of poor health compared to the non-energy poor population, without exception. 

**Figure 2:**
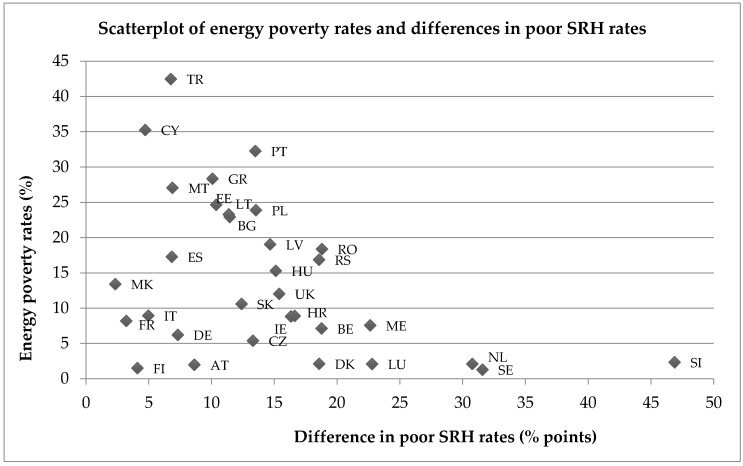
Scatterplot of energy rates and differences in poor SRH rates between energy poor and non-energy poor populations across 32 European countries.

The most striking aspect of Figure 1 is the magnitude of the difference in poor health prevalence between the non-energy poor and the energy poor populations within each country. Indeed, Slovenia, Sweden and the Netherlands have the largest differences, ranging from 46.9 percentage points in Slovenia, to 30.8 percentage points in the Netherlands, despite being relatively equal societies. To explore this relationship further, Figure 2 plots the percentage point differences in poor SRH rates between energy poor and non-energy poor populations against the overall energy poverty rates for each country, revealing interesting trends. The full country name for each two-letter country code can be found in Table A1 in Appendix A. In a number of instances larger disparities in poor SRH can be found in countries with lower rates of energy poverty, which appears to be somewhat paradoxical. However, it is important to note that this trend may partly be influenced by the smaller sub-sample sizes found in countries with low rates of energy poverty. Further analysis of larger datasets is necessary to confirm this initial observation. 

The logistic regression results for the association between energy poverty and poor SRH are displayed in Table 2. The results show that households living in energy poverty have a significantly increased risk of reporting poor SRH in the majority of European countries, after adjustments for socioeconomic factors. The highest risks of poor SRH are found in Slovenia, Sweden, and Ireland, where energy poor households are 26.32, 7.12 and 6.52 times more likely to report poor SRH compared to non-energy poor households. 

### 4.2. Poor Emotional Well-Being and Energy Poverty 

**Figure 3:**
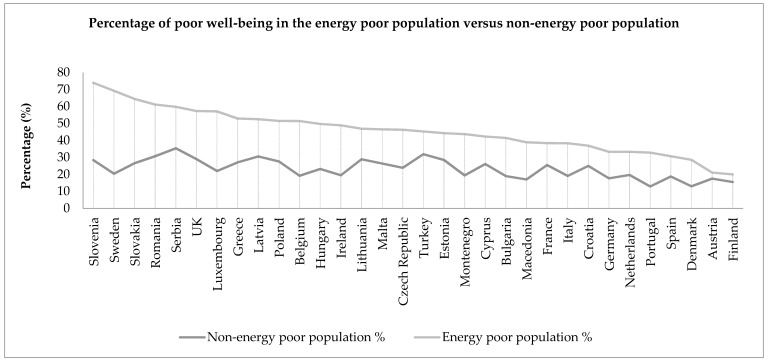
Line graph showing the prevalence of poor emotional well-being among the energy poor and non-energy poor populations across 32 European countries.

As before, the difference in mean scores for the WHO-5 index were compared for individuals in energy poor homes and those not. Well-being was found to be lower for individuals living in energy poverty than those who were not in all countries except Finland. Mann–Whitney U tests showed these differences were statistically significant in 30 of the study countries, the exceptions being Finland and the Netherlands. Subsequently, prevalence rates were calculated for the incidence of poor emotional well-being (WHO-5 index score ≤ 50) in the non-energy poor and energy poor populations, as displayed in Figure 3. 

**Figure 4:**
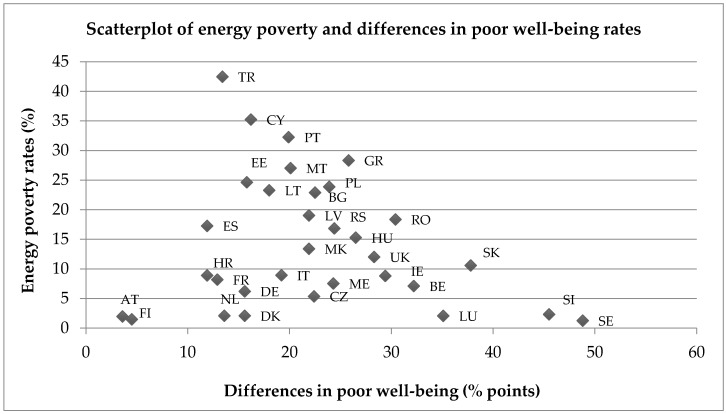
Scatterplot of energy rates and differences in poor well-being rates between energy poor and non-energy poor populations across 32 European countries.

As was found with poor health, there is an uneven distribution of poor emotional well-being across Europe, with the highest overall prevalence rates found within Central and Eastern Europe, and Turkey. In all countries, the prevalence of poor well-being is higher within the energy poor population than the non-energy poor population, with Slovenia and Sweden again exhibiting a large difference in prevalence between groups, along with Slovakia. The scatterplot in Figure 4 depicts the percentage point differences in poor well-being rates between energy poor and non-energy poor populations against the overall energy poverty rates for each country.

**Figure 5:**
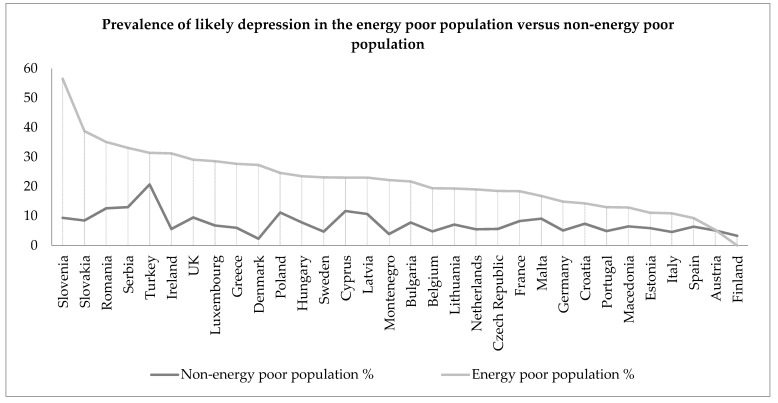
Line graph showing the prevalence of likely depression among the energy poor and non-energy poor populations across 32 European countries.

In Figure 5, the prevalence rates for likely depression (WHO-5 index score ≤ 28) are plotted for the energy poor and non-energy poor populations in each country. Here we find a higher prevalence of likely depression among the energy poor populations in most countries, although the within-country differences in prevalence are substantially smaller than for SRH and poor well-being, particularly for Sweden. The Finnish results are somewhat anomalous, however, this is unsurprising as the difference in mean WHO-5 scores is not statistically significant. As was mentioned earlier, some countries have relatively small sub-samples, thus necessitating future analysis of larger datasets to confirm initial observations in this paper.

**Figure 6:**
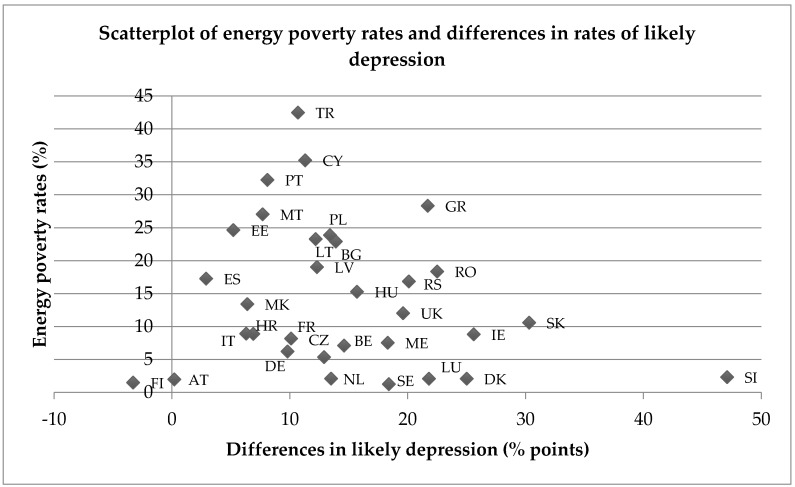
Scatterplot of energy rates and differences in likely depression rates between energy poor and non-energy poor populations across 32 European countries.

As before, the within-country differences in the rates of likely depression between energy poor and non-energy poor households are plotted against the overall energy poverty rates for each country, as can be seen in Figure 6. Similar geographical groupings emerge, although they are less pronounced than for poor SRH and well-being.

The combined logistic regression results in Table 3 establish that the risks of experiencing poor emotional well-being, as well as likely depression, are greater among energy poor households than non-energy poor households across nearly all European countries. In terms of poor well-being, the highest odds ratio can be found in Sweden (7.84), Greece (3.80), and Ireland (3.76), which broadly reflects the results for poor SRH, where energy poor households in Slovenia, Sweden and Ireland were at a greater risk of experiencing poor SRH than their non-energy poor counterparts. Somewhat similarly, the highest odds ratio for likely depression can be found in Denmark (34.58), Slovenia (19.93), and Ireland (6.42).

## 5. Discussion

The starting point of this paper was to add to the highly limited knowledge base that exists on energy poverty at the European-level by providing evidence about the relationship between general health, well-being, and energy poverty. While the findings presented in this paper do not and cannot replicate the nuanced evidence base that links health and energy poverty (for example, the impact of low temperatures on vulnerable groups or those with specific health conditions described in the background section), a clear relationship between energy poverty and poor health and well-being has been established using self-reported measures. The findings presented in this paper have shown that across the majority of 32 countries in Europe, the energy poor population is statistically more likely to report poor health and emotional well-being than the non-energy poor population, with a higher incidence of bad and very bad SRH, poor emotional well-being, and likely depression. In broad terms, the findings from this paper are in line with existing literature that looks at the relationship between SRH and energy poverty [60] and mental health and energy poverty [26,27,37].

This study has indicated a highly uneven situation across Europe, and has identified a number of differences between countries that is worthy of further discussion. There are a variety of possible explanations for these geographical variations, including: differences in national policy priorities and investment for health and energy (including the impact of government interventions in energy bills and taxation); housing conditions and domestic energy efficiency standards; and the specific policies of energy suppliers (including payment methods and tariffs), all of which are compounded by spatial and distributional effects. 

One of the most striking findings in our study has been the magnitude of the difference in poor health and well-being prevalence between the energy poor and non-energy poor populations within each country. In particular, Slovenia, Sweden, the Netherlands, Slovakia, Luxembourg and Denmark have among the largest differences in health and well-being outcomes between the energy poor/non energy poor populations, despite being countries with higher levels of income equality [79], and lower rates of energy poverty [20]. This partly confirms earlier work from 2004 by Healy [60], who found that the most notable difference in SRH status in EU14 occurred in the Netherlands, with a difference of 14.3 percentage point (versus our 2012 figure of 30.8 percentage points). The trend observed in our study appears to be somewhat paradoxical, however, there is evidence to suggest that experiencing deprivation in countries with lower income inequality and where there is a low overall risk of energy poverty occurring, results in worsened SRH due to the role of individual-level relative deprivation. Mishra and Carleton [71] outline the relative deprivation hypothesis, which suggests that “inequality affects health at the individual level through negative consequences of social comparison” [71]. These comparisons can have negative impacts to people’s emotions, behaviour, mental and physical health [80]. The results of a community study in Canada demonstrated that subjective feelings of personal relative deprivation are statistically associated with poorer physical and mental health outcomes [71]. Similarly, in a longitudinal study of the Swedish population, Åberg Yngwe et al. [81] show that relative deprivation is significantly associated with mortality, despite Sweden being “characterized by relatively small income inequalities and promoting values as egalitarianism and equality” [81]. 

Examining infrastructural and institutional changes at the national and regional level may also help to explain these findings. For instance, Tkalec and Živčič [82] note that energy poverty is becoming an increasing concern in Slovenia due to energy price rises outstripping increases to household incomes. The authors also highlight issues of energy degradation, with an increasing share of wood-based fuel in the energy mix [82], likely caused by the 137 per cent increase to the average retail price of heating oil between 2003 to 2011. Similarly, Sweden has seen significant and continued increases to the price of all energy sources since 1990 [83], and in particular to electricity heating costs, which has driven the expansion of district heating networks. Indeed by 2013, around 52 per cent of the Swedish population were supplied with district heating [84], bringing along with it limited control over internal temperatures and cost [85]. It has been noted by Lindén et al. [86] that this had led to a divergence in energy-related practices between households living in detached single dwellings, who pay for their heating costs directly to the energy supplier, and households in multi-occupancy buildings who normally have their heating included in their monthly rent, with the former heating their homes around 2 °C lower [86]. This rapidly unfolding energy landscape challenges existing conceptualisations of vulnerability in Europe and brings new potential issues, such as indoor air pollutants resulting from incomplete combustion of wood-based fuels [87].

Whilst these findings are limited by the measures of energy poverty, health and well-being used within the study (and indeed the available comparative data), it is the first pan-European study in over a decade that considers this relationship, with the findings pointing to an urgent need for additional in-depth research.

## 6. Conclusions

Within this exploratory study, we have found that across the majority of countries in Europe, the energy poor population is statistically more likely to report poor health and emotional well-being than the non-energy poor population, with a higher incidence of bad and very bad SRH, poor emotional well-being, and likely depression. Interestingly, we find the largest disparities in health and well-being levels between energy poor and non-energy poor households occur within relatively equal societies, such as Sweden and Slovenia. In terms of next steps for research, there is a clear need for further work to investigate why these apparent national differences exist, perhaps using statistical techniques such as cluster analysis, and political theory on the role of welfare state types. Additional analysis could incorporate further detail around national policy styles, energy prices and expenditure, and housing stock differences. In policy terms, this study lends support to the argument that in order to reduce the health inequalities associated with energy poverty, investment in energy efficiency schemes should be prioritised for energy poor households, in order to potentially realise reductions to public expenditure on health care [88]. However, consideration needs to be given to the funding mechanism used in order to avoid regressive effects. 

## Figures and Tables

**Table 1:** Prevalence rates (weighted) for key measures of energy poverty, health, and well-being, sorted by energy poverty rates. SRH: self-reported health status

Country	Unweighted Sample Size	Energy Poverty (%)	Poor SRH (%)	Poor Well-Being (%)	Likely Depression (%)
Sweden	1007	1.3	7.7	21.3	5.0
Finland	1020	1.5	7.0	15.6	3.2
Austria	1032	2.0	3.9	17.6	5.1
Denmark	1024	2.1	12.7	13.3	2.8
Luxembourg	1005	2.1	6.7	22.6	7.2
Netherlands	1008	2.1	9.9	20.0	5.8
Slovenia	1008	2.3	9.8	29.8	10.4
Czech Republic	1012	5.4	11.7	25.2	6.5
Germany	3055	6.2	8.9	18.7	5.8
Belgium	1013	7.1	8.9	21.7	5.9
Montenegro	1000	7.5	9.9	21.7	5.5
France	2270	8.2	6.6	26.7	9.1
Ireland	1051	8.8	4.4	22.2	7.9
Croatia	1001	8.9	13.5	25.9	8.0
Italy	2250	8.9	5.6	21.0	5.2
Slovakia	1000	10.6	10.6	30.5	11.8
UK	2252	12.0	11.0	32.6	11.9
Macedonia	1006	13.4	6.5	19.9	7.3
Hungary	1024	15.3	16.0	27.3	10.2
Serbia	1002	16.9	16.4	39.6	16.4
Spain	1512	17.3	5.6	20.9	7.0
Romania	1542	18.4	17.2	36.6	16.8
Latvia	1009	19.0	16.1	34.8	13.1
Bulgaria	1000	22.9	11.1	24.5	11.0
Lithuania	1134	23.3	20.8	33.3	10.0
Poland	2262	23.9	13.0	33.4	14.4
Estonia	1002	24.7	14.9	33.1	7.6
Malta	1001	27.1	3.2	31.3	11.1
Greece	1004	28.3	8.1	34.3	12.0
Portugal	1013	32.3	14.9	19.3	7.5
Cyprus	1006	35.2	3.3	31.8	15.6
Turkey	2035	42.5	8.6	37.6	25.4

**Table 2:** Logistic regression results for the association between energy poverty and poor SRH. Odds ratio (OR) and 95% confidence intervals (95% CI).

Country	OR	95% CI
Austria	3.19	0.52–19.65
Belgium	**3.11**	**1.50–6.43**
Bulgaria	**2.10**	**1.24–3.53**
Croatia	1.33	0.67–2.64
Cyprus	2.22	1.00–5.15
Czech Republic	1.99	0.92–4.30
Denmark	2.81	0.96–8.24
Estonia	1.20	0.76–1.91
Finland	1.03	0.18–5.89
France	1.10	0.61–1.99
Germany	**1.70**	**1.04–2.78**
Greece	**2.44**	**1.31–4.56**
Hungary	**2.43**	**1.41–4.19**
Ireland	**6.52**	**3.10–13.71**
Italy	1.51	0.77–2.95
Latvia	**1.73**	**1.11–2.70**
Lithuania	**1.70**	**1.17–2.46**
Luxembourg	3.72	0.83–16.60
Macedonia	1.59	0.70–3.61
Malta	**5.63**	**2.11–15.05**
Montenegro	**3.82**	**1.77–8.22**
Netherlands	**4.70**	**1.76–12.59**
Poland	**1.63**	**1.17–2.27**
Portugal	**2.23**	**1.30–3.80**
Romania	**1.91**	**1.31–2.77**
Serbia	1.70	0.98–2.95
Slovakia	**2.32**	**1.18–4.60**
Slovenia	**26.32**	**4.76–145.68**
Spain	**2.80**	**1.48–5.29**
Sweden	**7.12**	**1.62–31.40**
Turkey	**1.91**	**1.28–2.85**
UK	**2.18**	**1.46–3.25**

Results which differ significantly from zero (at *p* < 0.05) are in bold typeface.

**Table 3:** Logistic regression results for the association between energy poverty and poor well-being, and energy poverty and likely depression. Odds ratio (OR) and 95% confidence intervals (95% CI).

Country	Poor Well-Being		Likely Depression	
	Odds Ratio	95% CI	Odds Ratio	95% CI
Austria	0.91	0.27–3.12	1.05	0.13–8.16
Belgium	**3.68**	**2.00–6.76**	**3.19**	**1.41–7.23**
Bulgaria	**2.93**	**1.95–4.39**	**2.69**	**1.63–4.45**
Croatia	1.09	0.61–1.94	1.06	0.47–2.39
Cyprus	1.36	0.94–1.98	1.51	0.95–2.41
Czech Republic	**2.15**	**1.16–4.00**	**2.63**	**1.10–6.29**
Denmark	2.35	0.81–6.77	**34.58**	**8.36–143.01**
Estonia	**1.49**	**1.03–2.15**	1.44	0.81–2.57
Finland	1.71	0.43–6.71	0.00	0.00–0.00
France	**1.59**	**1.13–2.24**	**2.01**	**1.28–3.14**
Germany	**1.95**	**1.35–2.81**	**2.29**	**1.39–3.78**
Greece	**3.80**	**2.56–5.65**	**4.56**	**2.71–7.68**
Hungary	**2.49**	**1.63–3.82**	**2.69**	**1.55–4.66**
Ireland	**3.76**	**2.28–6.19**	**6.42**	**3.48–11.84**
Italy	**1.78**	**1.19–2.67**	1.47	0.76–2.84
Latvia	**2.26**	**1.56–3.27**	**2.00**	**1.25–3.21**
Lithuania	**1.97**	**1.44–2.70**	**2.57**	**1.65–4.00**
Luxembourg	1.38	0.38–5.04	2.18	0.44–10.78
Macedonia	**3.36**	**2.05–5.48**	**2.53**	**1.20–5.34**
Malta	**2.01**	**1.32–3.05**	**1.79**	**1.01–3.18**
Montenegro	**2.09**	**1.09–4.02**	**3.91**	**1.66–9.19**
Netherlands	1.72	0.65–4.52	2.67	0.75–9.43
Poland	**2.10**	**1.64–2.68**	**1.67**	**1.23–2.28**
Portugal	**3.00**	**1.91–4.72**	**2.30**	**1.20–4.40**
Romania	**2.62**	**1.91–3.58**	**2.54**	**1.80–3.59**
Serbia	**2.02**	**1.25–3.27**	**1.99**	**1.18–3.35**
Slovakia	**2.58**	**1.50–4.46**	**3.08**	**1.65–5.76**
Slovenia	**3.36**	**1.02–11.03**	**19.93**	**5.29–75.09**
Spain	**1.74**	**1.15–2.64**	1.79	0.93–3.45
Sweden	**7.84**	**1.62–38.02**	3.39	0.64–18.00
Turkey	**1.65**	**1.32–2.05**	**1.47**	**1.15–1.87**
UK	**2.64**	**1.91–3.64**	**2.76**	**1.89–4.03**

Results which differ significantly from zero (at *p* < 0.05) are in bold typeface.

## Appendix A

**Table A1:** Two-letter country codes.

Country Name	Two-Letter Country Code
Austria	AT
Belgium	BE
Bulgaria	BG
Croatia	HR
Cyprus	CY
Czech Republic	CZ
Denmark	DK
Estonia	EE
Finland	FI
France	FR
Germany	DE
Greece	GR
Hungary	HU
Ireland	IE
Italy	IT
Latvia	LV
Lithuania	LT
Luxembourg	LU
Macedonia	MK
Malta	MT
Montenegro	ME
Netherlands	NL
Poland	PL
Portugal	PT
Romania	RO
Serbia	RS
Slovakia	SK
Slovenia	SI
Spain	ES
Sweden	SE
Turkey	TR
United Kingdom	UK
